# Integrating multi-omics and epiphytic microbial communities to decipher the spatiotemporal dynamics of flower color dynamic regulation in *Hibiscus mutabilis*

**DOI:** 10.3389/fpls.2026.1803034

**Published:** 2026-04-23

**Authors:** Shengwen Tang, Xi Chen, Jiao Ma, Pei Tu, Xiaoqing Shi, Guo Chen

**Affiliations:** Chengdu Park City Botanical Science Research Institute, Chengdu Botanical Garden, Chengdu, Sichuan, China

**Keywords:** epiphytic microbiome, flower color change, mechanism, plant–microbe interaction, spatiotemporal regulation

## Abstract

The dynamic change of flower color is a key trait for plant environmental adaptation and pollinator attraction, yet its spatiotemporal regulatory mechanisms remain poorly understood. *Hibiscus mutabilis* L., known for its remarkable diurnal color-changing phenomenon, provides an ideal model for deciphering the dynamic regulation of flower color. This study integrated metabolomics, transcriptomics, and epiphytic microbial community analyzes to systematically compare the spatiotemporal differences between petals and the flower base of *H. mutabilis*. Metabolomic analysis revealed that differential metabolites between petals and the flower base were primarily enriched in pathways such as glycolysis and glutathione metabolism. Differences between morning and afternoon in the flower base were concentrated in flavonoid biosynthesis and amino acid metabolism pathways. Transcriptomic analysis identified MYB114-like was significantly upregulated in the afternoon, with its expression co-enriched with genes from the plant hormone signaling and MAPK pathways, suggesting it participates in the regulatory mechanism of environmental signal responses. Microbial community analysis showed a significant increase in the relative abundance of Actinomycetota in the flower base from morning to afternoon. Functional prediction suggested that these microbes might be involved in processes such as redox metabolism and nucleotide degradation. This study reveals a multidimensional regulatory network involving metabolism, transcription, and microbes governing the dynamic color change in *H. mutabilis*, from the perspective of spatial heterogeneity and plant-microbe interactions, providing novel insights into the mechanisms of flower color formation and the adaptability of ornamental plants.

## 1 Introduction

Flower color is not a static ornament, but a highly dynamic and complex biological trait finely regulated by genetic programs and environmental signals. It plays a central role in plant reproduction, serving as a visual signal to attract pollinators, thereby directly influencing species perpetuation (Reverté et al., 2016; Trunschke et al., 2021; Liu et al., 2024). Furthermore, flower color affects a plant’s adaptive capacity to abiotic stressors, its ornamental value, and consequently its economic worth in horticulture and agriculture (Grotewold, 2006; Zhao and Tao, 2015; Liu et al., 2022). Most flower colors are formed by three major classes of pigments: anthocyanins, carotenoids, and betalains. Their optical properties and final perceived color are not solely determined by pigment composition, but are further influenced by a suite of cofactors, including vacuolar pH, the presence of copigments, and even subcellular cell morphology, which affects light scattering. Recent studies indicate that flavonoid-based pigments, particularly anthocyanins, are uniquely suited for dynamic modulation (Markham et al., 2000; Stavenga et al., 2020). The chromophore of these organisms is highly sensitive to intracellular pH and redox states, allowing rapid, non-enzymatic color shifts in response to environmental cues. Moreover, the biosynthetic pathway of anthocyanins is tightly linked to environmental sensing networks, enabling transcriptional and post-translational regulation of pigment production in real-time under changing abiotic and biotic conditions. As anthropogenic activities and climate change persistently alter light regimes, temperature patterns, and pollinator community structures, a comprehensive understanding of the mechanisms underlying color generation and dynamic regulation has become crucial. This knowledge is vital not only for fundamental ecological and evolutionary research but also for guiding the sustainable cultivation, breeding, and conservation of ornamental plants in rapidly changing environments.

For a long time, research on the mechanisms of flower color formation has primarily focused on the biosynthesis, accumulation, and spatial distribution of pigments such as anthocyanins, flavonoids, and carotenoids within petals (Yeo and Moyroud, 2025). Significant diurnal color changes within a single flower, exemplified by the famous and dramatic color-changing phenomenon in *Hibiscus mutabilis*, challenge this static perspective and offer an exceptional model to dissect how external environmental signals are rapidly perceived, transduced, and converted into intracellular biochemical responses over a short timescale. Traditional pigment chemistry analysis can describe the quantitative outcomes of these changes but struggles to elucidate the complete, interconnected regulatory network driving this dynamic process. Critical questions remain largely unexplored frontiers in plant biology. First, do differentiated regulatory strategies exist among different floral organs? The flower is a complex structure with functionally distinct parts. The base is involved in nutrient transport and structural support, while the petal limb is specialized for display. Their metabolic and transcriptional landscapes are likely heterogeneous, yet most studies homogenize petals for analysis. Second, and more intriguingly, do the microbial communities colonizing petal surfaces participate in and influence this dynamic process? The phyllosphere, and specifically the anthosphere, is now recognized as a vibrant ecosystem where microbes interact with plant chemistry. The potential for these microbes to modulate or respond to rapid physiological changes like color shift is virtually unknown. Addressing these spatial and biotic interaction dimensions is essential for a holistic understanding of floral trait dynamics.

Among diverse floral color changes, rapid physiological color shifts occurring within a single flowering period are particularly rare and scientifically valuable. *H. mutabilis*, a classic ornamental tree widely used in landscaping and urban greening, is renowned for its striking diurnal color change (Shang et al., 2020; Ding et al., 2022). Its petals transition distinctly from white to pink and finally to deep red following a clear diurnal rhythm. This intriguing phenotypic shift makes *H. mutabilis* an ideal model system for studying dynamic pigment metabolism and its regulatory network. Early studies indicated that the color transition is associated with *de novo* synthesis of anthocyanins, especially cyanidin-3-sambubioside, accompanied by increased phenylalanine ammonia-lyase activity in the petals (Amrhein and Frank, 1989). Furthermore, at the base of the flower, the local accumulation of free anthocyanins can cause distinct marks to appear at the base, revealing strong spatial heterogeneity in pigment patterning within a single flower (Lowry, 1971; Zhao, 2023). Recent integrated metabolomic and transcriptomic analyzes of different diurnal color stages in *H. mutabilis* revealed coordinated regulation between flavonoid biosynthetic genes and anthocyanin accumulation during the transition from white to pink (Zhu et al., 2023; Shi et al., 2024). However, these studies primarily analyzed whole petal tissues without explicitly distinguishing between distal petals and the flower base, nor did they decipher the diurnal changes specific to the flower base itself.

Meanwhile, diverse leaf-marginal microbial communities inhabit plant surfaces, influencing host physiology, stress responses, and signaling characteristics (Meyer et al., 2022; Mahmoudi et al., 2024; Yang et al., 2025). Microbial communities attached to flower surfaces interact with petals, nectar, and pollen (Aleklett et al., 2014). Recent studies indicate that flavonoids and other secondary metabolites in plants contribute to the establishment of leaf-marginal microbial communities, while colonizing microorganisms regulate the host’s metabolic capacity, defense capabilities, and signal transduction (Liu et al., 2020; Bashir et al., 2022). Nevertheless, research on floral color change has rarely focused on the petal surface microbiome. The potential links between dynamic pigment accumulation, the local metabolite environment, and microbial community assembly remain largely unexplored.

Therefore, this study employed an integrated analysis of metabolomics, transcriptomics, and microbial communities, focusing on deciphering color-associated differences between petals and the flower base of *H. mutabilis*, with particular emphasis on comparing the flower base between morning and afternoon. This work provides novel insights into the complex regulatory mechanisms underlying dynamic flower color formation in *H. mutabilis* and reveals the potential functional role of the floral microbiome in plant secondary metabolism.

## 2 Materials and methods

### 2.1 Sample collection

**Figure 1:**
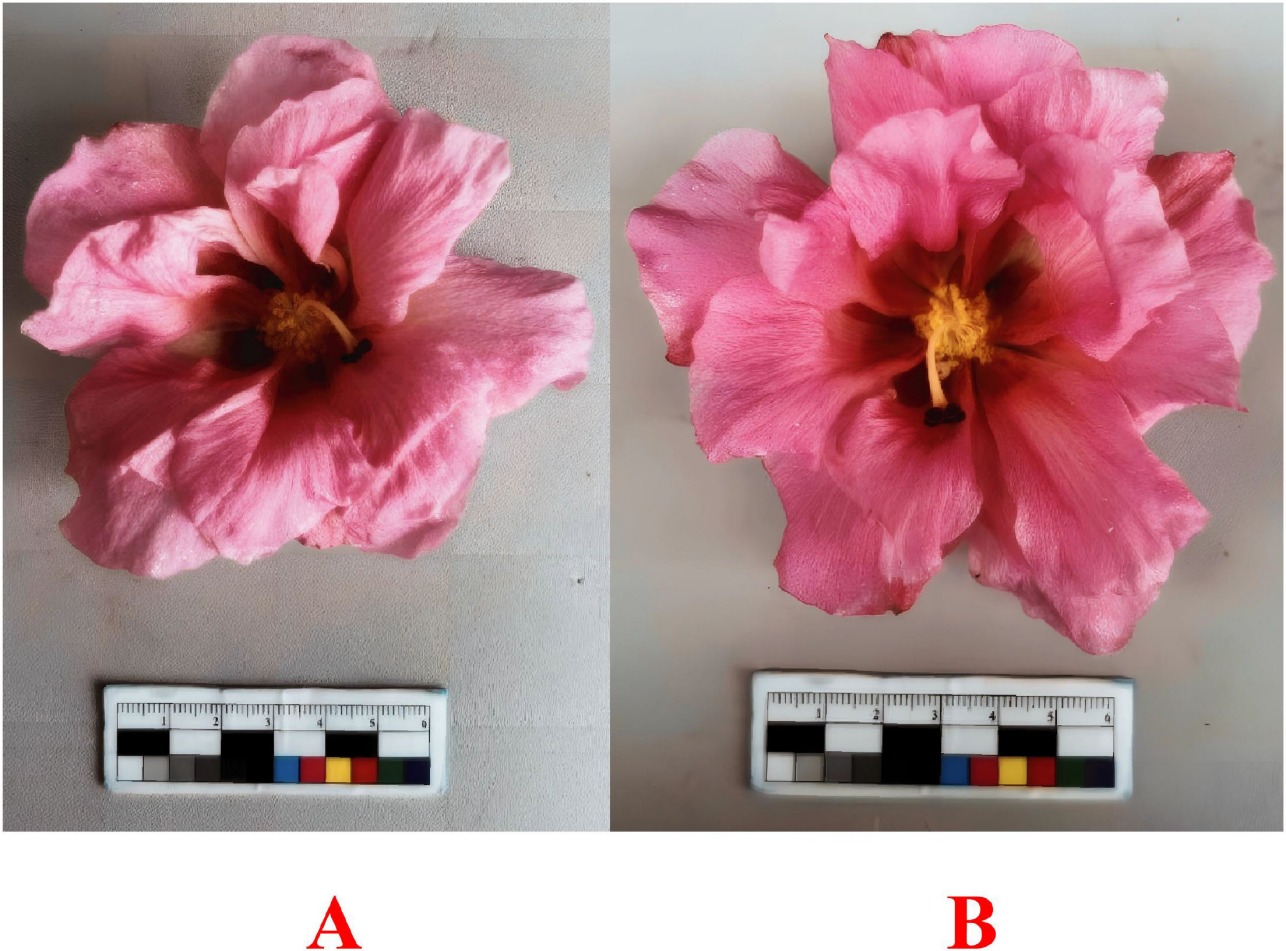
Flowers collected at different times. **(A)** Flower collected at 10:00 AM. **(B)** Flower collected at 5:00 PM.

The *H. mutabilis* flowers used in this study were grown under natural conditions and collected from the Chengdu Botanical Garden (104°10’ E, 30°40’ N). Sampling was conducted at the fully open flower stage. Flowers from the same plant were collected at 10:00 AM and 5:00 PM, respectively (**Figure 1**). Petal tissue samples collected in the morning were designated HB, flower base tissue collected in the morning was designated JM, and flower base tissue collected in the afternoon was designated JA. Each group consisted of three biological replicates. Samples were immediately frozen in liquid nitrogen and stored at **−**80 °C for subsequent analysis.

### 2.2 Metabolite extraction and analysis

Petals and flower base tissues were cryopreserved in liquid nitrogen and ground into fine powder. Approximately 100 mg of tissue sample was placed in an EP tube and extracted with 500 μL of 80% methanol. The mixture was vortexed, incubated on ice for 5 min, then centrifuged at 15,000 g and 4 °C for 20 min. A portion of the supernatant was diluted with mass spectrometry-grade water to a methanol concentration of 53%. Centrifuge at 15,000 g for 20 min at 4 °C, collect the supernatant, and inject for LC-MS analysis (Xiong et al., 2025b). Samples were injected onto a Hypersil Goldcolumn (100×2.1 mm, 1.9μm) using a 12-min linear gradient at a flow rate of 0.2 mL/min. The eluents for the positive and negative polarity modes were eluent A (0.1% formic acid in water) and eluent B (methanol). The solvent gradient was set as follows: 2% B, 1.5 min; 2-85% B, 3 min; 85-100% B, 10 min; 100-2% B, 10.1 min; 2% B, 12 min. The Q ExactiveTM HF mass spectrometer (Thermo Fisher, Germany) was operated in positive/negative polarity mode with a spray voltage of 3.5 kV, a capillary temperature of 320 °C, a sheath gas flow rate of 35 psi, and an auxiliary gas flow rate of 10 L/min. The S-lens RF level was set to 60, and the auxiliary gas heater temperature was set to 350 °C. Implement quality control to ensure system stability. Convert raw data files to mzXML format using ProteoWizard. Perform peak extraction and quantification with XCMS, aligning peaks across samples based on retention time, mass-to-charge ratio, and other parameters. Then, based on adduct ions and setting mass deviation to 10 ppm, a comparison was made between these data and the self-built high-quality secondary spectrum database (NovoMetDB) to obtain results for metabolite identification. Metabolites were annotated using public databases (KEGG, HMDB, and LIPIDMaps) and quantified based on normalized peak areas. Differentially expressed metabolites (DEMs) were identified using the threshold |log2(FoldChange)| ≥ 1.5 and FDR < 0.05. Pathway enrichment analysis was performed using KEGG annotations to identify significantly affected metabolic pathways.

### 2.3 Transcriptome sequencing and analysis

Total RNA from petal and flower base tissues was extracted using a plant RNA extraction kit (Omega Bio-Tek, Norcross, GA, USA) following the manufacturer’s instructions. RNA quality was assessed using an Agilent 2100 Bioanalyzer. After library qualification, sequencing was performed on an Illumina platform to generate paired-end reads. The obtained RNA-seq data were processed and assembled using Trinity (v2.15.1) (Haas et al., 2013). Redundant transcripts were aggregated using Corset based on the Trinity assembly (Davidson and Oshlack, 2014). The quality of the assembled transcripts was evaluated using BUSCO (Benchmarking Universal Single-Copy Orthologs). Gene function was annotated based on the Nr, Nt, Pfam, KOG/COG, Swiss-Prot, KEGG, and GO databases (Ashburner et al., 2000; Kanehisa and Goto, 2000; Boutet et al., 2007; Pruitt et al., 2007; Galperin et al., 2021; Mistry et al., 2021). Expression levels were quantified using FPKM. Differential expression genes (DEGs) were screened using the DESeq2 R package (1.42.0) with thresholds of padj ≤ 0.05 and |log2(foldchange)| ≥ 1. GO enrichment analysis of DEGs was performed using the GOseq R package. Statistical enrichment of DEGs in KEGG pathways was tested using KOBAS 3.0 software.

### 2.4 DNA extraction and sequencing of floral epiphytic microorganisms

DNA extraction and 16S rRNA sequencing of microorganisms on the surface of petal and flower base samples were performed following standard protocols. Total genomic DNA was extracted from samples using a DNA extraction kit. The V3-V4 region of the bacterial 16S rRNA gene was amplified using primers 341F (CCTAYGGGRBGCASCAG) and 806R (GGACTACNNGGGTATCTAAT) (Xiong et al., 2025a). Each PCR mixture contained 15 µL Phusion High-Fidelity PCR Master Mix, 0.2 µM of each primer, and 10 ng of genomic DNA template. PCR conditions were: initial denaturation at 98 °C for 1 min, 30 cycles of denaturation at 98 °C for 10 s, annealing at 50 °C for 30 s, and extension at 72 °C for 30 s, final extension at 72 °C for 5 min. PCR products were purified using magnetic beads, pooled in equimolar amounts based on concentration, detected, and the target bands were recovered. Libraries were constructed, quantified using Qubit and qPCR, and sequenced on the NovaSeq 6000 platform after passing quality checks. Sequencing was performed by Novogene Co., Ltd. (Beijing, China).

## 3 Results

### 3.1 Metabolomic data analysis

Metabolites in all samples across the three groups were analyzed via metabolomics. A total of 3,920 metabolites were detected, including 560 lipids and lipid-like molecules, 351 organic acids and derivatives, 160 flavonoids, 110 fatty acids and conjugates, among others. PCA analysis revealed significant differences between the HB and JM groups, as well as between the JM and JA groups, with variations in metabolite content between these pairs (**Supplementary Figure 1**).

### 3.2 Differential metabolite analysis

**Figure 2:**
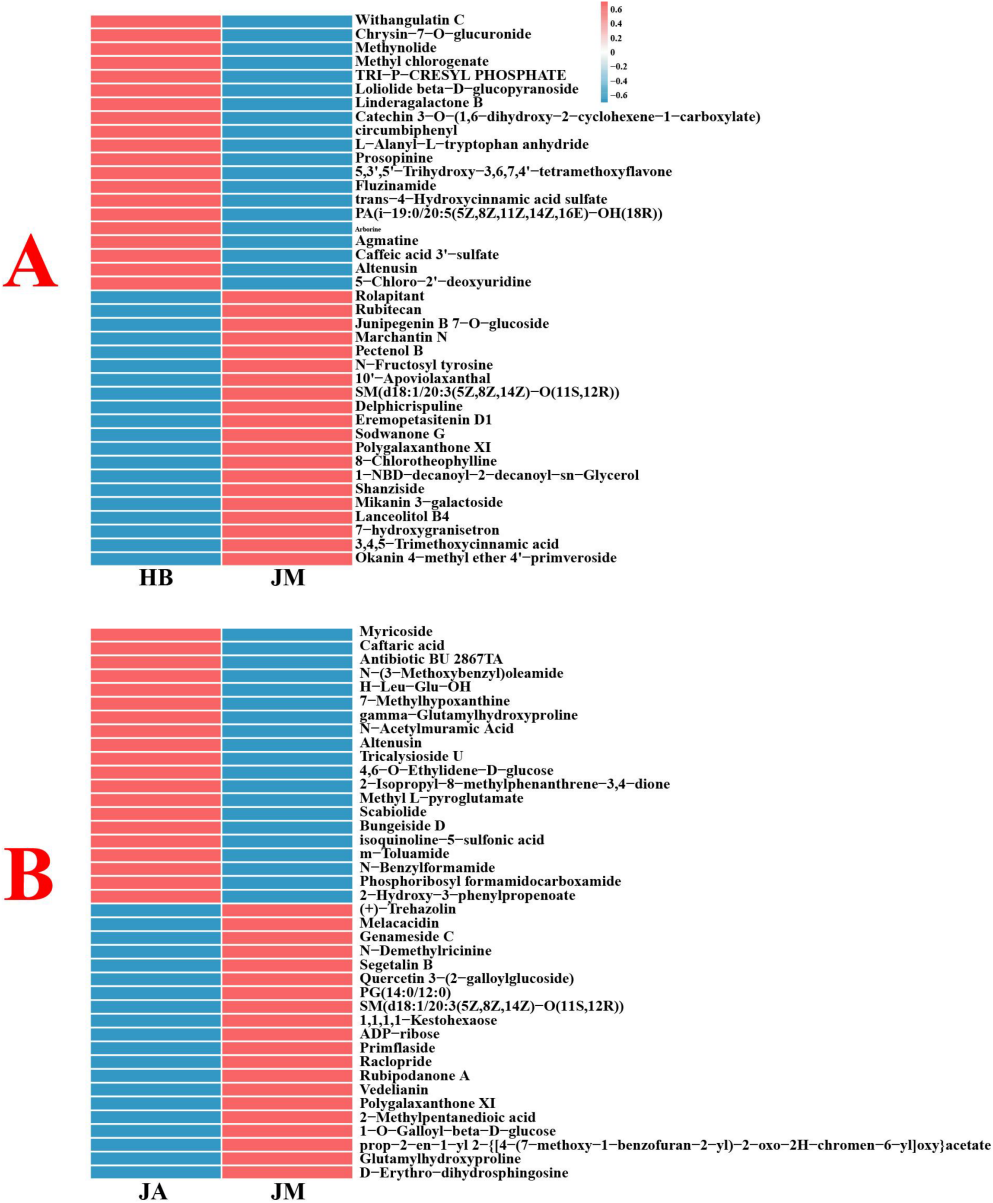
The top 40 DEMs with the most significant changes between HB and JM groups **(A)**, and between JA and JM groups **(B)**.

**Figure 3:**
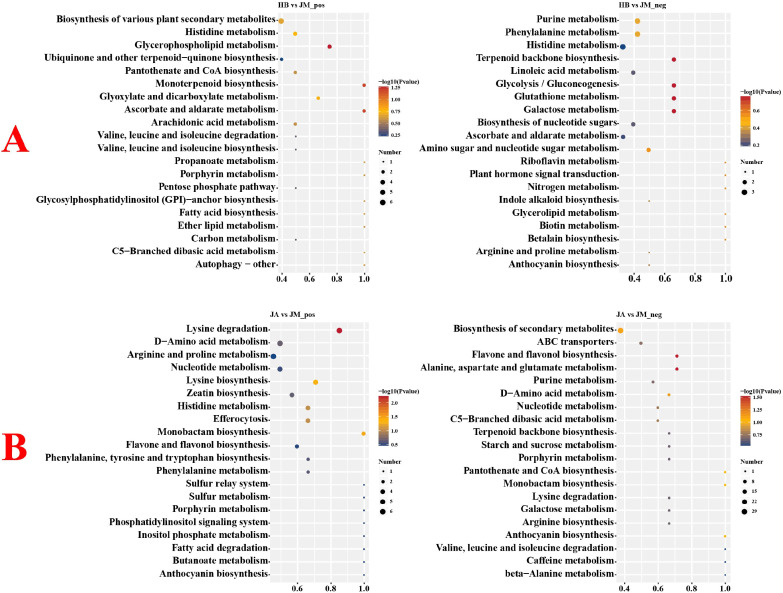
KEGG enrichment analysis bubble chart of DEMs between groups under positive and negative ion conditions. **(A)** KEGG enrichment analysis bubble chart between the HB and JM groups. **(B)** KEGG enrichment analysis bubble chart between the JA and JM groups.

Volcano plots were used to analyze metabolite changes between different groups (**Supplementary Figure 2**). Between HB and JM groups, 183 metabolites were upregulated and 582 downregulated in positive ion mode, while 95 were upregulated and 353 downregulated in negative ion mode. Between JA and JM groups, 173 metabolites were upregulated and 512 downregulated in positive ion mode, while 148 were upregulated and 169 downregulated in negative ion mode. Heatmaps compared the top 40 most significantly changed DEMs between groups (**Figure 2**). Between HB and JM, DEMs like Tri-p-cresyl phosphate, Pectenol B, Catechin 3-O-(1,6-dihydroxy-2-cyclohexene-1-carboxylate), and Sodwanone G showed the most significant changes. Between JM and JA, DEMs such as N-Acetylmuramic Acid, SM(d18:1/20:3(5Z,8Z,14Z)-O(11S,12R)), Caftaric acid, and N-Demethylricinine changed most significantly. KEGG enrichment analysis revealed that between HB and JM, pathways like Glycolysis/Gluconeogenesis, Glutathione metabolism, Galactose metabolism, and Glycerophospholipid metabolism were significantly affected (**Figure 3**). Between JM and JA, pathways such as Lysine degradation, Flavone and flavonol biosynthesis, and Alanine, aspartate and glutamate metabolism were significantly affected.

### 3.3 Transcriptomic data analysis

Transcriptome sequencing yielded a total of 58.75 Gb of data from all samples, with an average of 6.53 Gb per sample. After quality control, a total of 391,676,942 clean reads were obtained, averaging 43,519,660 clean reads per sample. The average Q20 and Q30 of clean sequences were 99.37% and 97.02%, respectively, with an average GC content of 43.31%.

### 3.4 Differential expression gene analysis

**Figure 4:**
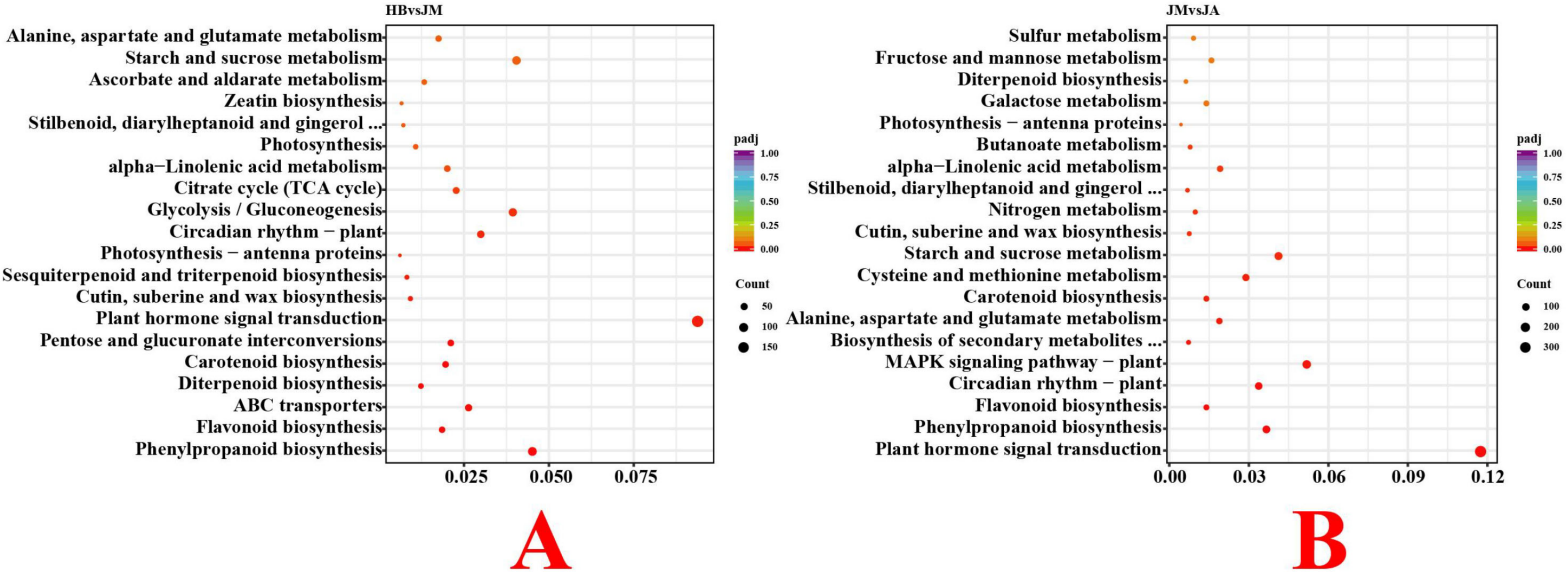
KEGG enrichment analysis bubble plots of DEGs between two groups. **(A)** KEGG enrichment analysis bubble plot between the HB group and the JM group. **(B)** KEGG enrichment analysis bubble plot between the JM group and the JA group.

Under the screening criteria of padj ≤ 0.05 and |log2FoldChange| ≥ 1, a total of 10,414 DEGs were identified between HB and JM groups, with 4,996 upregulated and 5,418 downregulated (**Supplementary Figure 3**). Between JM and JA groups, 15,998 DEGs were identified, with 8,283 upregulated and 7,715 downregulated. Comparison of the top 20 most differentially expressed genes revealed that between HB and JM, genes included myb domain protein 5, transcription factor MYB114-like, Lysozyme 1, chitinase A, and laccase-15-like. Between JM and JA, genes included *Arabidopsis thaliana* gibberellin 2-oxidase 4, vacuolar invertase 2, carotenoid cleavage dioxygenase 4, salt tolerance zinc finger, and trehalose-6-phosphate phosphatase G. GO enrichment analysis showed that DEGs in HB compared to JM were mainly enriched in Molecular Function (MF) terms such as catalytic activity, oxidoreductase activity, and catalytic activity, acting on RNA (**Supplementary Figure 4**). DEGs between JM and JA were also enriched in MF terms like catalytic activity, transferase activity, and oxidoreductase activity. KEGG enrichment analysis indicated that between HB and JM, DEGs were significantly enriched in pathways including Plant hormone signal transduction, Phenylpropanoid biosynthesis, and Glycolysis/Gluconeogenesis (**Figure 4**). Between JM and JA, DEGs were significantly enriched in pathways such as Plant hormone signal transduction, MAPK signaling pathway-plant, and Starch and sucrose metabolism.

### 3.5 16S rRNA sequencing data analysis

The structure of epiphytic microbial communities in samples from the three groups was analyzed via 16S rRNA amplicon sequencing. Initially, a total of 1,670,934 raw sequences were obtained from all samples, averaging 185,659 raw sequences per sample. After merging and filtering, 1,598,399 effective tags were finally obtained, averaging 177,600 effective tags per sample. The average Q20 and Q30 of clean sequences were 98.36% and 94.26%, respectively, with an average GC content of 55.31%.

### 3.6 Classification and abundance of microbial communities

**Figure 5:**
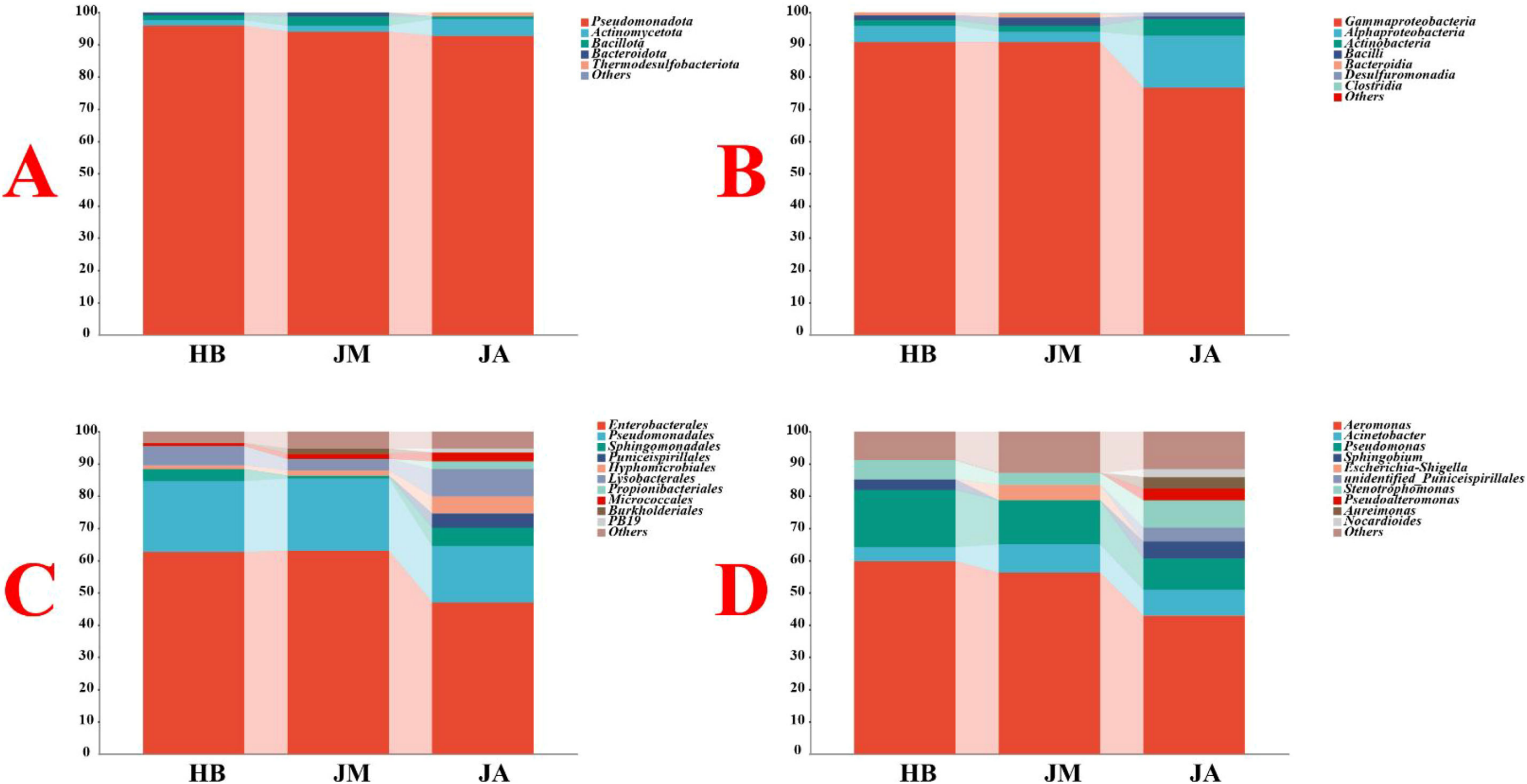
Abundance distribution of bacteria on petal surfaces at the phylum **(A)**, class **(B)**, order **(C)**, and genus **(D)** levels.

A total of 5 phyla, 7 classes, 20 orders, 29 families, and 32 genera were identified from samples across the three groups. At the phylum level, Pseudomonadota was significantly more abundant than other bacterial phyla, accounting for 94.24% (**Figure 5A**). This was followed by Actinomycetota and Bacillota, accounting for 2.95% and 1.74%, respectively. At the class level, Gammaproteobacteria was significantly more abundant, accounting for 86.08%, followed by Actinomycetota and Bacillota at 8.17% and 2.95%, respectively (**Figure 5B**). Notably, the abundance of Actinomycetota in the JM group was significantly lower compared to the JA group. At the order level, Enterobacterales was most abundant at 57.56%, followed by Pseudomonadales and Sphingomonadales at 20.75% and 3.35%, respectively (**Figure 5C**). At the genus level, *Aeromonas* was most abundant at 53.01%, followed by *Pseudomonas* and *Acinetobacter* at 13.65% and 7.10%, respectively (**Figure 5D**).

### 3.7 Alpha and beta diversity analysis

**Figure 6:**
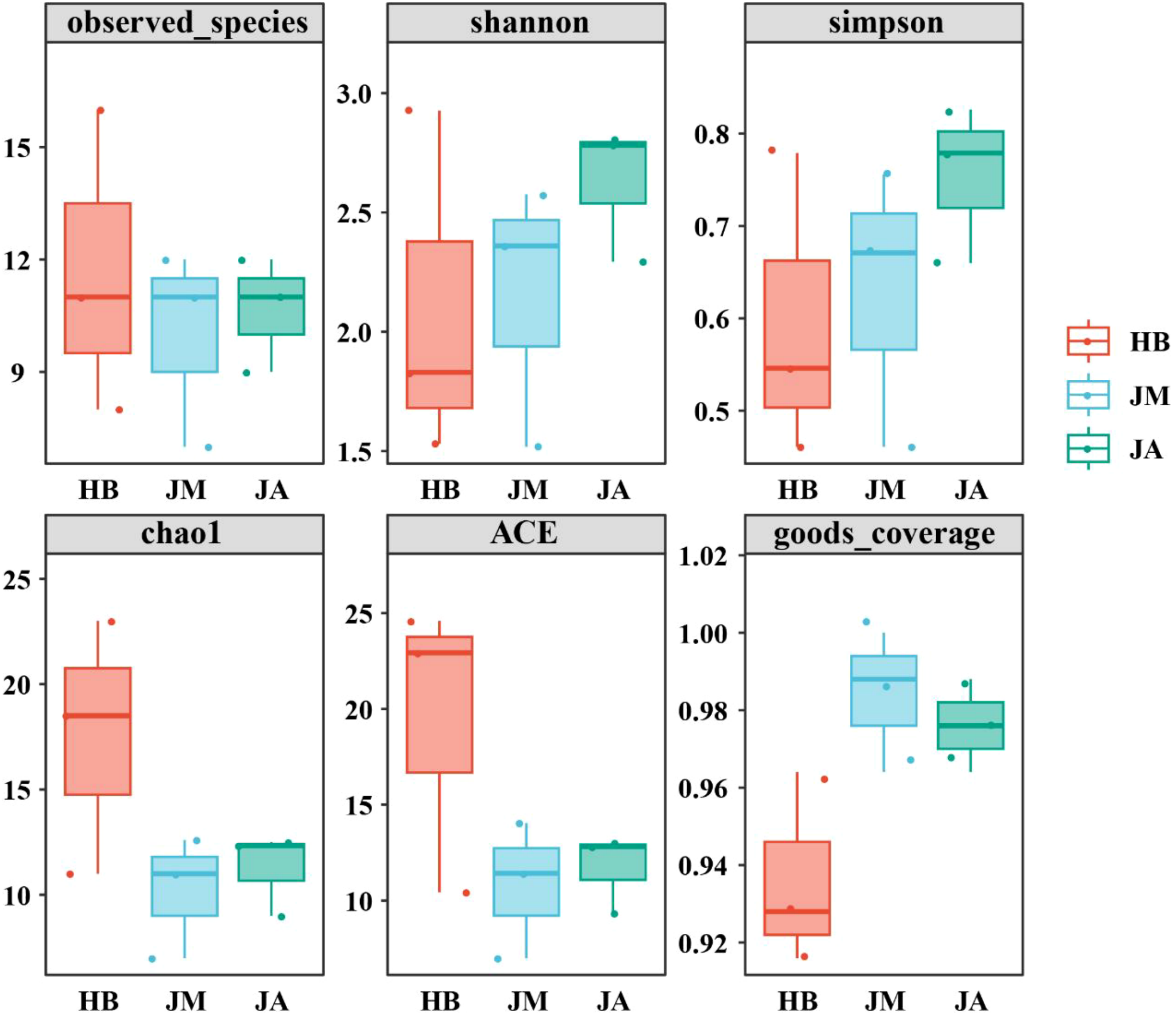
Box plot of α diversity index for bacteria on petal surfaces.

**Figure 7:**
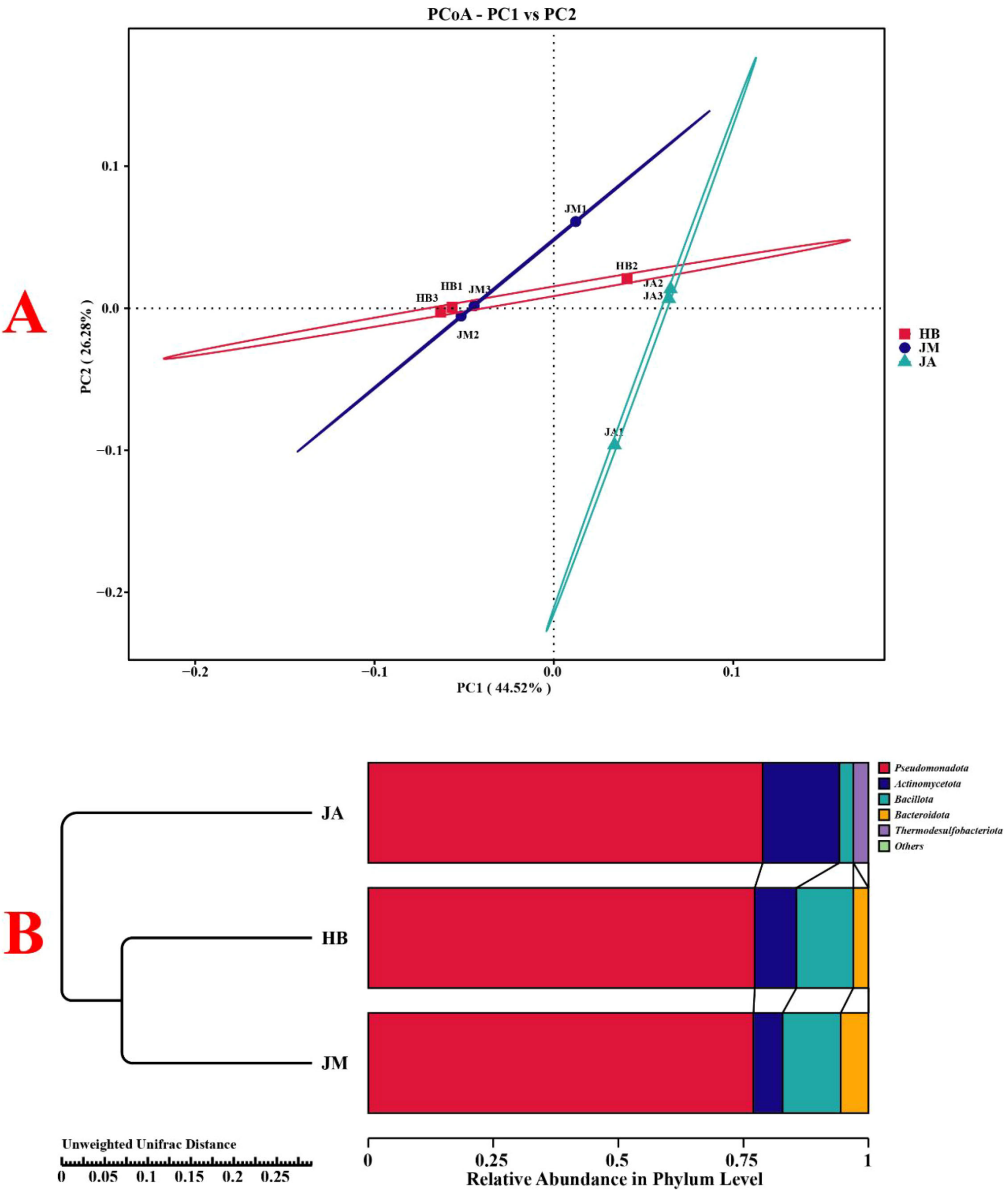
Analysis of β-diversity in petal surface bacterial communities using PCoA **(A)** and assessment of structural changes in petal surface bacterial communities based on NMDS analysis and unweighted UniFrac distances **(B)**.

Indices used to assess species diversity and richness within sample groups are shown in **Figure 6**. For the observed species index, variation among the three groups was minimal. For the Shannon and Simpson indices, the JM group was higher than HB, and the JA group was higher than JM. For the Chao1 and ACE indices, both JM and JA groups were lower than HB, with little change between JM and JA. Notably, the goods coverage index was significantly higher in JM compared to HB, with little change between JM and JA. PCoA analysis revealed relatively small changes in bacterial community structure among the three groups (**Figure 7A**). The PC1 axis accounted for 44.52% of the differences in epiphytic bacteria on flowers, while the PC2 axis accounts for 26.28%. The difference between HB and JM was relatively small, while the difference between JM and JA was relatively larger. Furthermore, analysis based on weighted UniFrac distances showed that JA and JM exhibited the greatest community structure difference, while HB and JM were most similar (**Figure 7B**).

### 3.8 Functional prediction analysis

Based on COG database analysis, compared to HB, the JM group showed significantly decreased levels of functions like Multidrug efflux pump subunit AcrB (COG0841) and Outer membrane receptor for ferric coprogen and ferric-rhodotorulic acid (COG4773), while levels of ABC-type multidrug and LPS transport system (COG1132) and ABC-type polar amino acid transport system (COG1126) were significantly increased (**Supplementary Figure 5A**). Compared to JM, the JA group showed significantly increased levels of functions such as NAD(P)-dependent dehydrogenase (COG1028) and Enoyl-CoA hydratase/carnithine racemase (COG1024), while levels of functions like 23S rRNA C2501 and tRNA U34 5’-hydroxylation protein RlhA/YrrN/YrrO (COG0826) and 3-deoxy-D-arabino-heptulosonate 7-phosphate (DAHP) synthase (COG0722) were significantly decreased (**Supplementary Figure 5B**). Based on pathways database analysis, no significant functional changes were found between HB and JM. Compared to JM, the JA group showed significantly increased levels of functions like adenosine nucleotides degradation II (SALVADEHYPOX-PWY) and urate biosynthesis/inosine 5’-phosphate degradation (PWY-5695), while levels of functions like pyruvate fermentation to acetate and lactate II (PWY-5100) and superpathway of L-aspartate and L-asparagine biosynthesis (ASPASN-PWY) were significantly decreased (**Supplementary Figure 5C**).

### 3.9 Metabolism-transcription association analysis

In the morning-collected petal and floral base samples, although multiple DEMs and DEGs were enriched in metabolic pathways such as Glycerophospholipid metabolism, no DEMs or DEGs were found to be significantly enriched in the same metabolic pathway. However, in the flower base samples collected in the morning and afternoon, we identified 5 DEMs and 59 DEGs that were jointly enriched in the Alanine, aspartate, and glutamate metabolism pathway (**Supplementary Figure 6**). As shown in the figure, the expression of asparagine synthase (6.3.5.4) was significantly reduced, while that of asparaginase (3.5.1.1) was significantly increased, ultimately leading to a significant decrease in L-aspartate expression. Simultaneously, omega-amidase (3.5.1.3) expression was significantly reduced, while L-aspartate oxidase (1.4.3.16) expression was significantly increased, ultimately leading to a significant decrease in oxaloacetate expression.

### 3.10 Correlation analysis

**Figure 8:**
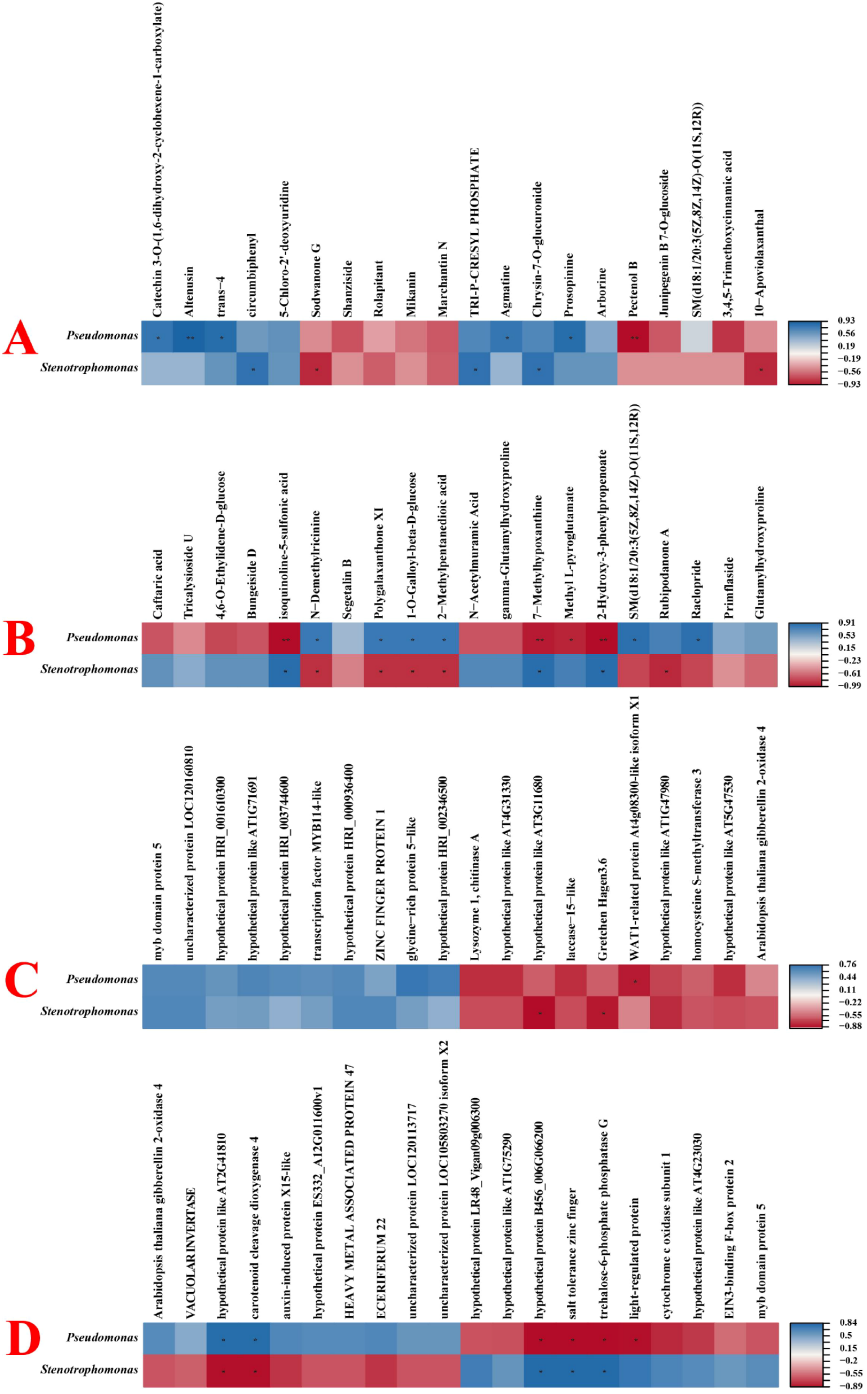
Correlation analysis between floral epiphytic microbial communities and differentially expressed metabolites and genes. **(A)** Correlation analysis between floral epiphytic microbial communities and differentially expressed metabolites in HB and JM groups. **(B)** Correlation analysis between floral epiphytic microbial communities and differentially expressed metabolites in JM and JA groups. **(C)** Correlation analysis between floral epiphytic microbial communities and differentially expressed genes in HB and JM groups. **(D)** Correlation analysis between floral surface microbial communities and differentially expressed genes in the JM group and JA group. An asterisk (*) indicates a significant correlation, and two asterisks (**) indicate a highly significant correlation.

**Figure 8** shows the correlations between the epiphytic microbial communities on flowers and DEMs and DEGs. Between the HB group and the JM group, *Pseudomonas* exhibited the most significant positive correlation with Altenusin and the most significant negative correlation with Pectenol B. *Stenotrophomonas* showed significant positive correlations with circumbiphenyl, TRI-P-CRESYL PHOSPHATE, and Chrysin-7-O-glucuronide, while exhibiting significant negative correlations with Sodwanone G and 10-Apoviolaxanthal. Between the JM group and the JA group, *Pseudomonas* showed significant positive correlations with DEMs such as N-Demethylricinine and Polygalaxanthone XI, while exhibiting significant negative correlations with DEMs like isoquinoline-5-sulfonic acid and 7-Methylhypoxanthine. *Stenotrophomonas* showed significant positive correlations with DEMs such as isoquinoline-5-sulfonic acid and 7-Methylhypoxanthine, while exhibiting significant negative correlations with DEMs like N-Demethylricinine and Polygalaxanthone XI. Between the HB group and the JM group, *Pseudomonas* showed a significant negative correlation with WAT1-related protein At4g08300-like isoform X1. *Stenotrophomonas* showed a significant negative correlation with hypothetical protein like AT3G11680 and Gretchen Hagen3.6. Between the JM group and the JA group, *Pseudomonas* showed a significant positive correlation with hypothetical protein like AT2G41810 and carotenoid cleavage dioxygenase 4, while exhibiting a significant negative correlation with hypothetical protein B456_006G066200 and salt tolerance zinc finger among DEGs. *Stenotrophomonas* showed significant positive correlations with hypothetical protein B456_006G066200 and salt tolerance zinc finger DEGs, while exhibiting significant negative correlations with hypothetical protein like AT2G41810 and carotenoid cleavage dioxygenase 4.

## 4 Discussion

By integrating metabolomics, transcriptomics, and microbial community analysis, this study systematically deciphered the spatiotemporal regulatory mechanisms underlying flower color evolution in *H. mutabilis*, clarifying the potential regulatory roles of organ-specific metabolism, transcriptional networks, and epiphytic microbial communities. This work addresses gaps in existing research concerning spatial heterogeneity and plant-microbe interactions.

Our findings demonstrate that the spatiotemporal differences in *H. mutabilis* flower color stem from a finely coordinated, multi-layered regulatory program involving distinct metabolic priorities and transcriptional reprogramming in different floral organs over time. The metabolomic data paint a clear picture of functional specialization. Differential metabolites between petals and the morning flower base were primarily enriched in fundamental pathways such as glycolysis and glutathione metabolism. This suggests that in the morning, the flower base is already primed with a high metabolic flux through central carbon metabolism, potentially generating energy and reducing power, as well as precursor molecules that feed into the shikimate and phenylpropanoid pathways upstream of flavonoid biosynthesis. The enrichment of glutathione metabolism further points to active management of redox balance, which is critical as the later burst of flavonoid and anthocyanin synthesis can generate reactive oxygen species. In stark contrast, the differences between the morning and afternoon flower base itself shift dramatically towards specialized secondary metabolism, being concentrated in flavonoid biosynthesis and amino acid metabolism pathways. This temporal shift indicates a strategic reallocation of resources within the flower base as the day progresses. From preparing the foundational metabolic infrastructure in the morning to executing the specific biosynthetic program for pigments in the afternoon. This aligns perfectly with the organ’s hypothesized role as a nutrient transport hub and metabolic control center, dynamically supplying precursors and managing the biochemical environment for the synthesis occurring in both the base and the petals. Metabolomic data indicate that the rapid transition from pink to deep red in petals is primarily driven by substantial anthocyanin accumulation, especially through the activation of flavone and flavonol biosynthetic pathways. This mechanism is consistent with metabolic patterns observed in other color-changing species like *Nicotiana alata* and *Rhododendron liliiflorum* (Zheng et al., 2021; Zhang et al., 2023). However, spatial analysis revealed significant heterogeneity between petals and the flower base. The flower base, as a metabolic center for pigment synthesis, showed significantly higher enrichment in sugar metabolism and glutathione pathways compared to distal petals. This suggests the flower base may provide essential carbon skeletons and redox homeostasis to sustain high-intensity anthocyanin metabolism during the afternoon peak. Furthermore, carotenoid cleavage dioxygenase 4, identified as a DEG between morning and afternoon in the flower base, suggests carotenoid degradation may serve as a purging mechanism to enhance the visual purity of anthocyanin-derived red color, a regulatory strategy also reported in *Crocus* species (Nørbæk et al., 2002; Krstić et al., 2024).

The transcriptomic data provide the regulatory logic behind these metabolic observations. The significant enrichment of DEGs in plant hormone signal transduction and MAPK signaling pathway-plant in both spatial and temporal comparisons is particularly revealing (Lang et al., 2025). It emphasizes that the diurnal color change is not just a passive biochemical cascade, but rather an active, signal-mediated physiological response. The MAPK cascade, as a classical pathway, may convert external stimuli such as light quality, light intensity, and temperature changes triggered by diurnal cycles into intracellular phosphorylation events, ultimately regulating gene expression. Concurrently, the enrichment of hormone signaling pathways suggests a finely tuned integration mechanism between environmental perception and endogenous developmental programs. This pathway may involve hormones such as abscisic acid, jasmonic acid, or ethylene signaling molecules known to regulate anthocyanin accumulation under stress or developmental cues (Loreti et al., 2008). Within this signaling context, the identification of MYB114-like protein and myb domain protein 5 as central hubs is of paramount importance. MYB transcription factors, as regulators of the MBW (MYB-bHLH-WD40) complex, have been well-validated in numerous species (Smith and Rausher, 2011; Lloyd et al., 2017a; Li et al., 2024). MYB transcription factors are well-established master regulators of the phenylpropanoid pathway, often acting within the MBW complex to directly activate anthocyanin biosynthetic genes (Lloyd et al., 2017b). The precise temporal upregulation of these MYB genes in the flower base from morning to afternoon positions them as key molecular switches, potentially directly activated by the converging MAPK and hormone signaling pathways in response to midday environmental conditions. Their actions likely drive the observed upregulation of flavonoid biosynthetic genes and the subsequent accumulation of anthocyanins. Furthermore, the induction of a carotenoid cleavage dioxygenase in the afternoon flower base presents an intriguing additional layer of regulation. By promoting the degradation of carotenoids, the plant may be actively clearing competing pigments from the chemical palette, thereby enhancing the visual purity and saturation of the anthocyanin-derived red color. This is a sophisticated strategy to sharpen the visual signal for pollinators, as reported in other species like *Crocus* (Nørbæk et al., 2002).

At the transcriptomic level, we identified MYB114-like protein and myb domain protein 5 as central hubs in the regulatory network. In *H. mutabilis*, the temporal upregulation of these MYB proteins precisely corresponds to the transition from morning to afternoon, indicating they may respond to diurnal environmental signals like light intensity and temperature. The co-enrichment of MAPK signaling and plant hormone signal transduction pathways further suggests these MYB proteins do not act in isolation but are integrated into a broader environmental sensing and response network, mediating the rapid physiological state transition of petals.

The structural characteristics and functional predictions of the epiphytic microbial community revealed its potential involvement in the dynamic regulation of flower color. Although the overall microbial community composition on *H. mutabilis* was still dominated by Pseudomonadota, we observed a significant increase in the relative abundance of Actinomycetota in the flower base during the transition from morning to afternoon, alongside various changes in alpha diversity, beta diversity, and microbial functions. This shift suggests that rapid changes in the floral microenvironment, particularly alterations in environmental factors like flavonoid accumulation and potential fluctuations in vacuolar or surface pH, exert selective pressure on the microbial community (Compant et al., 2019). Conversely, these microbes may participate in shaping floral phenotypes by modulating surface chemistry or producing secondary metabolites that stabilize anthocyanins. The enrichment of microbial functions related to redox metabolism, nucleotide degradation, and transport systems in the afternoon-collected flower base indicates that epiphytic microbes may respond to and potentially regulate the changing chemical landscape associated with pigment accumulation. Flavonoids and related phenolics have been shown to shape plant microbiome composition and activity (Fu et al., 2022). Our data suggest that metabolic shifts during *H. mutabilis* color change may restructure microbial functions, thereby influencing local metabolite turnover or signaling microenvironments.

The limitations of this study lie in the fact that it only performed differential analysis and pathway enrichment, without establishing a prediction and validation system for target genes. Therefore, subsequent related studies can build upon these findings to conduct validation research on target genes and associated microorganisms.

## 5 Conclusion

Through integrated multi-omics analysis, this study systematically elucidated the spatiotemporal dynamics regulating the diurnal color change in *H. mutabilis*. First, significant metabolic and transcriptional heterogeneity was found between petals and the flower base. The flower base, acting as a precursor supply center for pigment synthesis, exhibited significantly heightened activity in sugar metabolism and antioxidant pathways in the afternoon. Second, the transcription factor MYB114-like was highly expressed in the afternoon, potentially driving anthocyanin accumulation by integrating light signals and hormone pathways. Finally, the structure of the epiphytic microbial community on the flower base underwent significant succession accompanying color change, with an increase in the relative abundance of Actinomycetota and a functional shift towards redox metabolism, suggesting microbial involvement in regulating the local microenvironment related to flower color. These findings not only deepen the understanding of the molecular mechanisms underlying dynamic flower color formation in *H. mutabilis* but also incorporate the epiphytic microbial community into the regulatory network framework, providing a crucial basis for future research on the role of plant-microbe interactions in floral physiology and ecological function.

## Data Availability

The datasets presented in this study can be found in online repositories. The names of the repository/repositories and accession number(s) can be found in the article/**Supplementary Material**.

## Supplementary material

Supplementary Figure 1 PCA between HB and JM groups **(A)**, and between JM and JA groups **(B)**.

Supplementary Figure 2 Volcano plots of DEMs in positive and negative ion modes between HB and JM groups **(A)**, and between JA and JM groups **(B)**.

Supplementary Figure 3 Volcano plot of DEGs between HB and JM groups **(A)**, and between JM and JA groups **(B)**.

Supplementary Figure 4 GO enrichment analysis between HB and JM groups **(A)**, and between JM and JA groups **(B)**.

Supplementary Figure 5 Analysis between HB and JM groups **(A)**, between JM and JA groups **(B)** based on the COG database, and between JM and JA groups based on the pathways database **(C)**.

Supplementary Figure 6 Metabolic pathways of alanine, aspartate, and glutamate. Green boxes indicate significantly downregulated differentially expressed genes; red boxes indicate significantly upregulated differentially expressed genes. Blue circles indicate significantly downregulated differentially expressed metabolites; yellow circles indicate significantly upregulated differentially expressed metabolites.
