# Supplementary material for: Integrating multi-omics and epiphytic microbial communities to decipher the spatiotemporal dynamics of flower color dynamic regulation in Hibiscus mutabilis
Source: Front Plant Sci. 2026 Apr 23;17:1803034. doi: 10.3389/fpls.2026.1803034 (PMC13149465; doi:10.3389/fpls.2026.1803034)
Supplement: Supplementary Figure 1 — PCA between HB and JM groups (A), and between JM and JA groups (B). [file DataSheet1.docx]

**Supplementary Materials**

The sequencing data from this study have been uploaded to the NCBI database, with BioProject accession numbers PRJNA1419700 and PRJNA1419732.


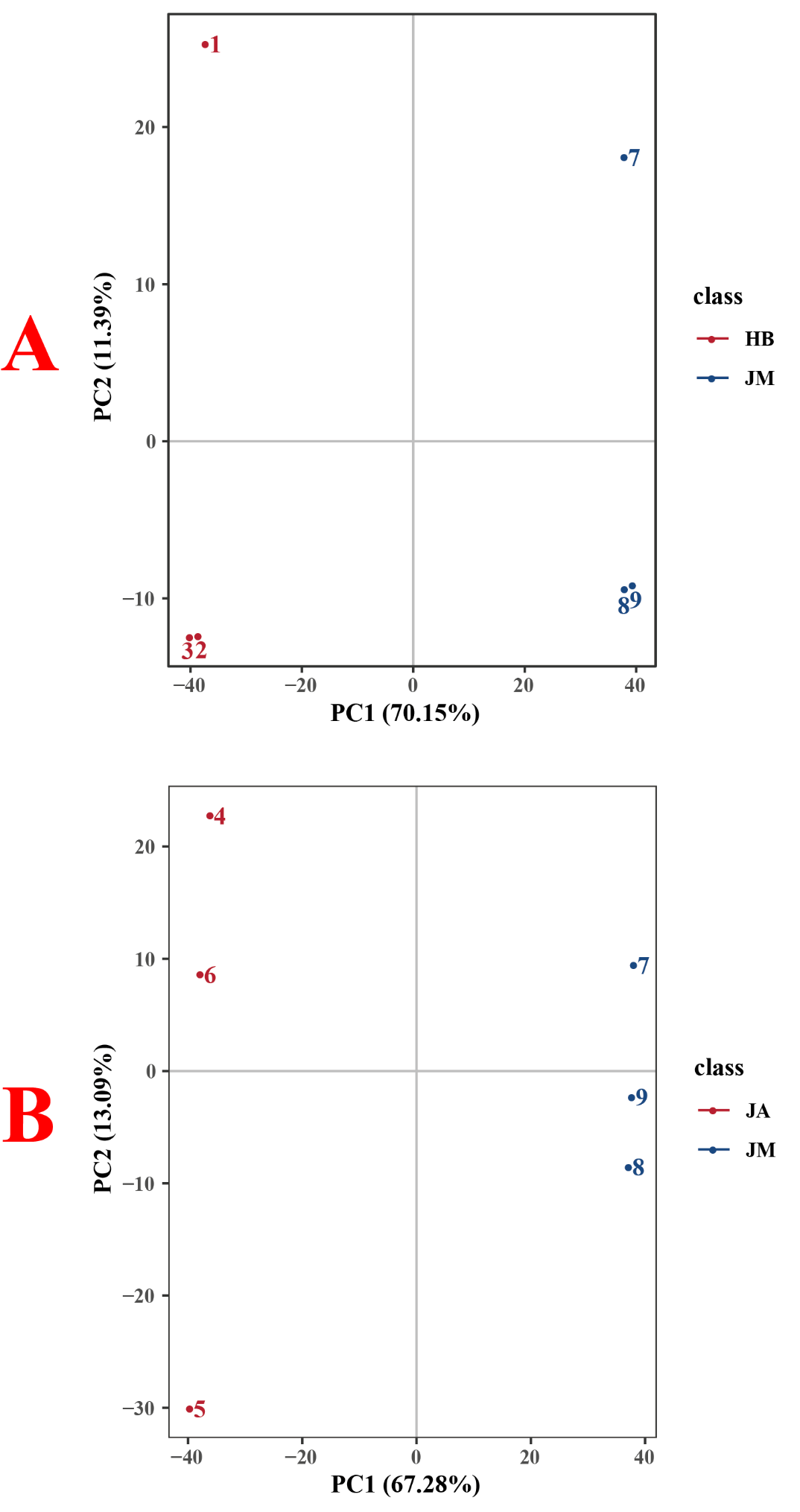


**Figure S1** PCA between HB and JM groups (A), and between JM and JA groups (B).


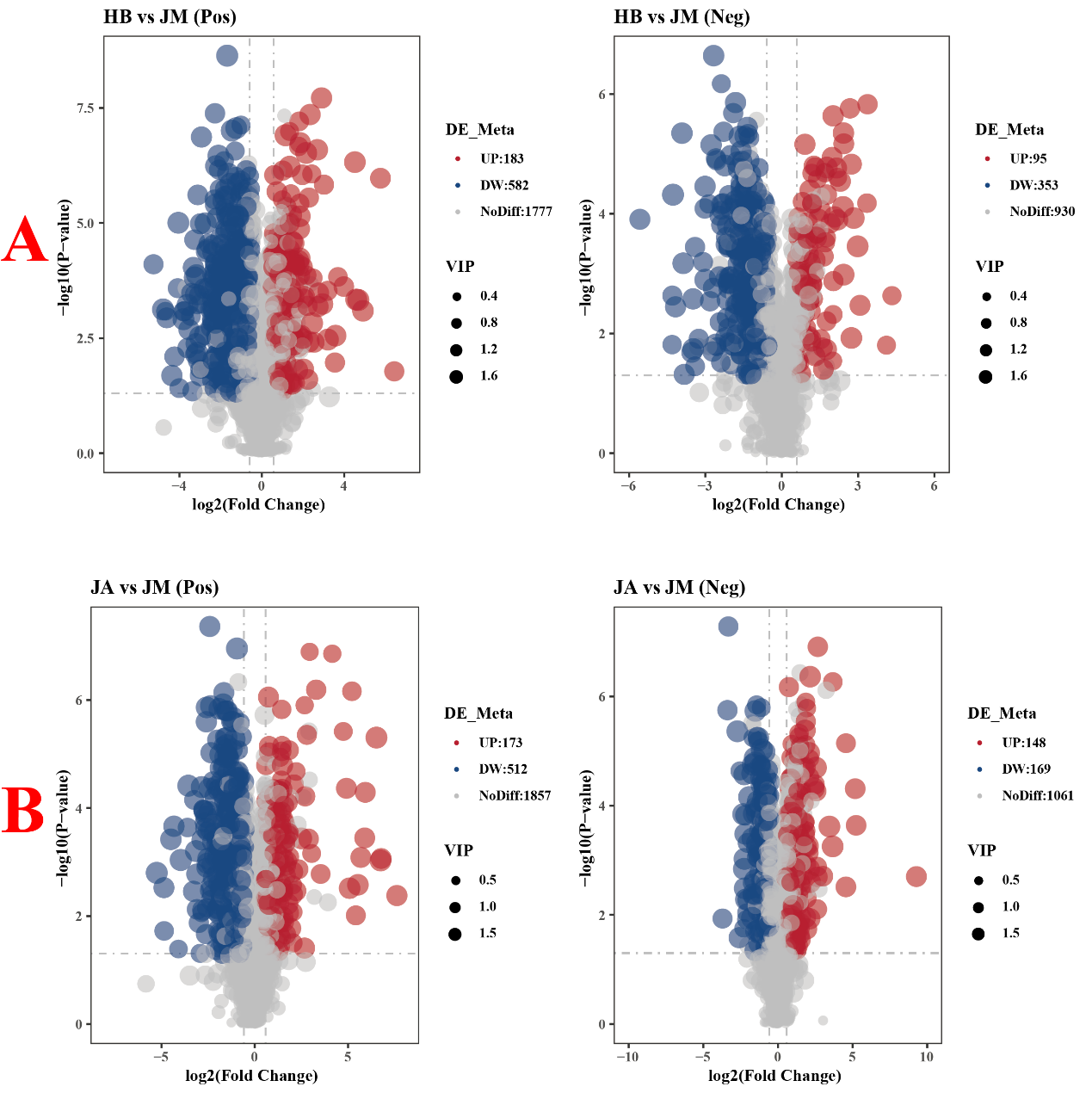


**Figure S2** Volcano plots of DEMs in positive and negative ion modes between HB and JM groups (A), and between JA and JM groups (B).


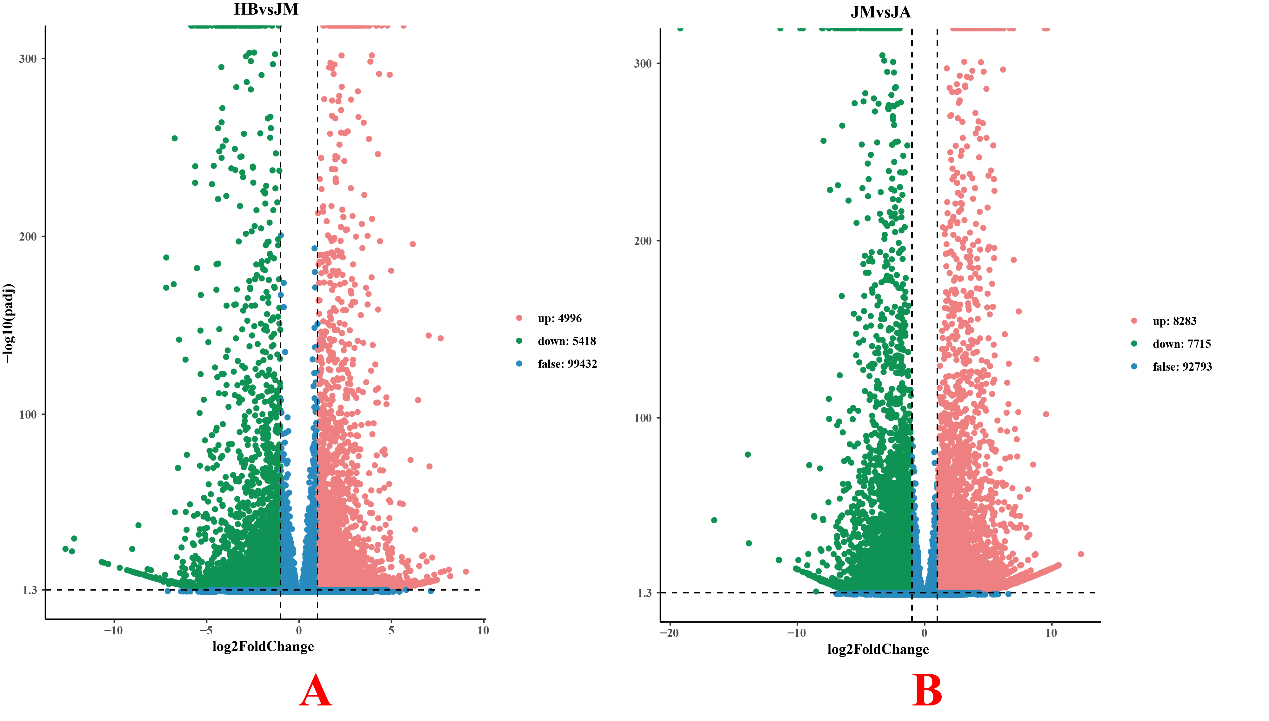


**Figure S3** Volcano plot of DEGs between HB and JM groups (A), and between JM and JA groups (B).


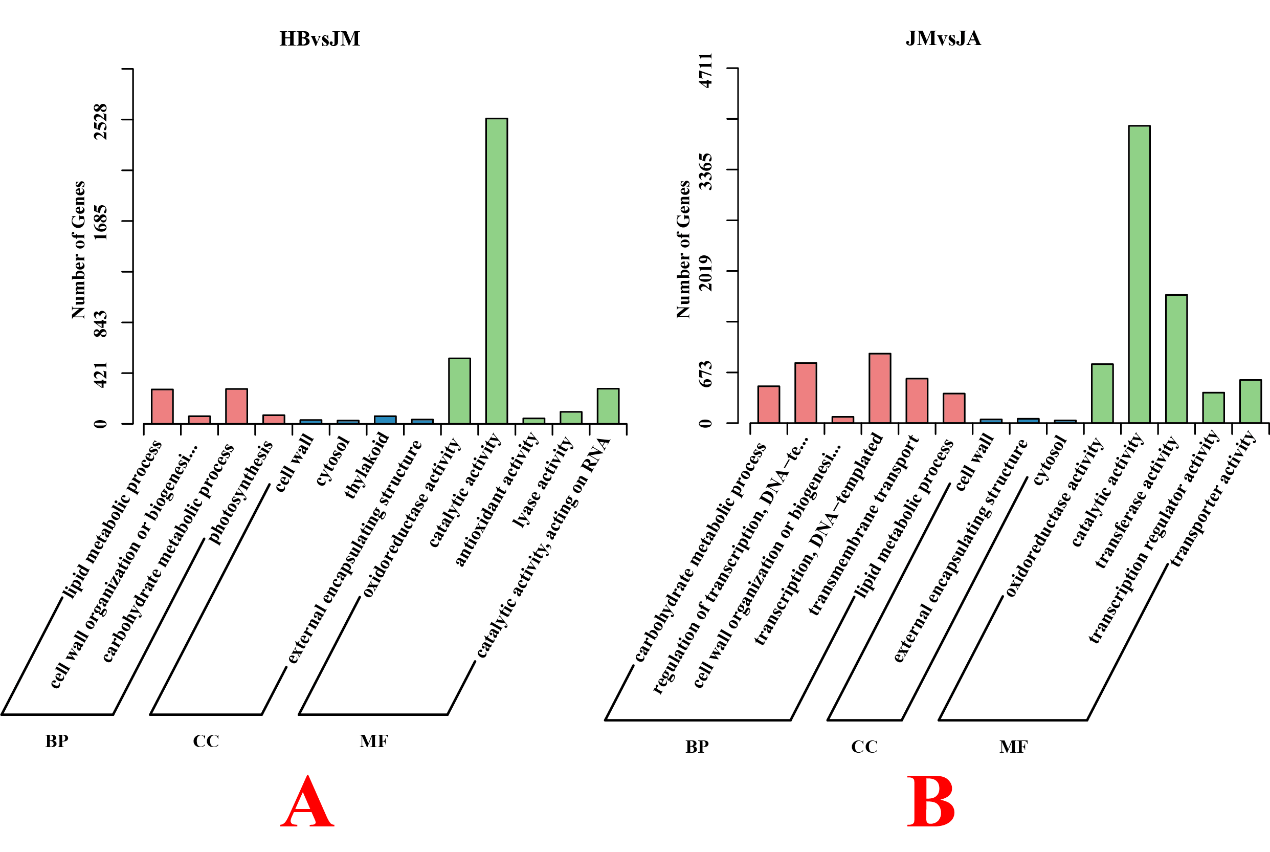


**Figure S4** GO enrichment analysis between HB and JM groups (A), and between JM and JA groups (B).


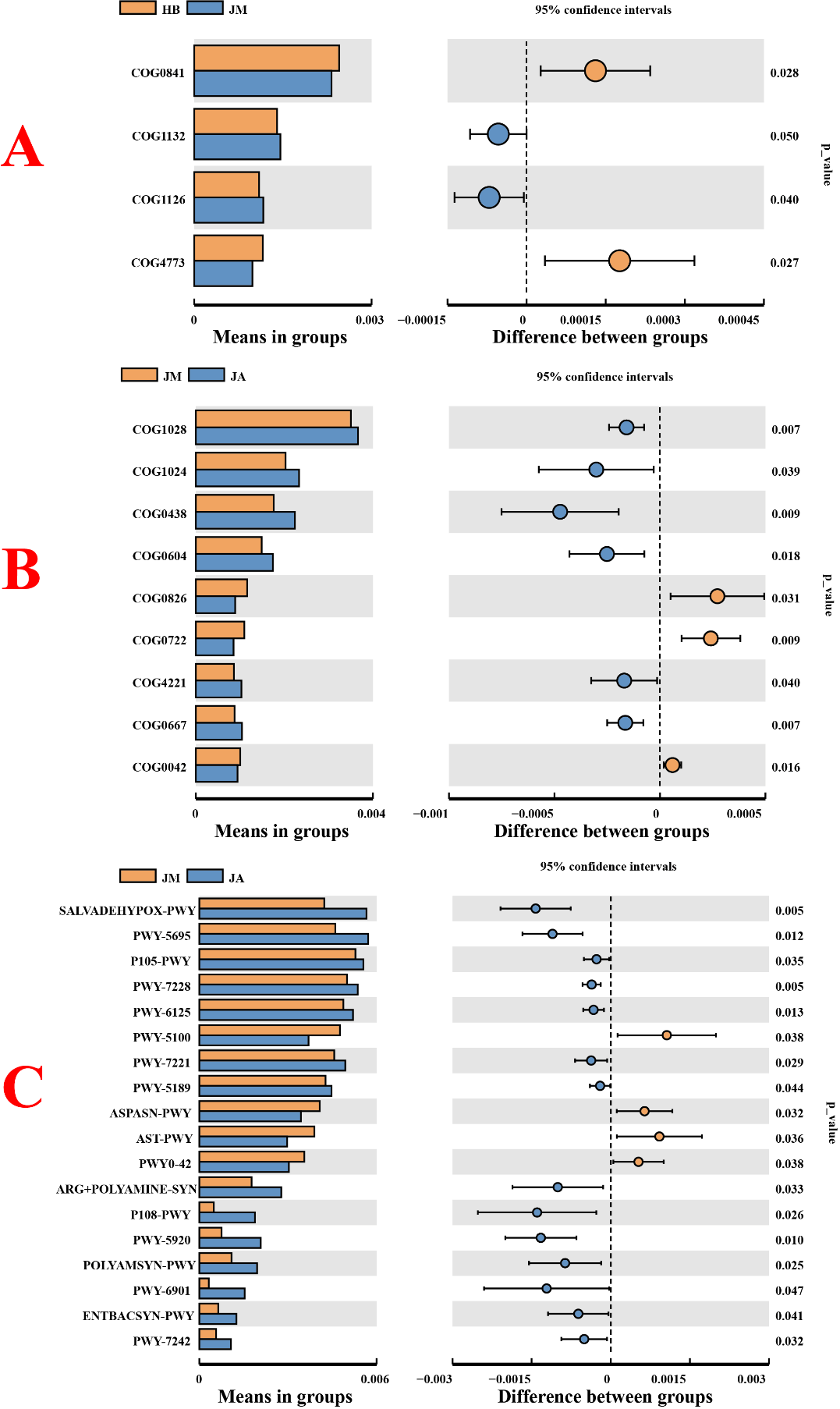


**Figure S5** Analysis between HB and JM groups (A), between JM and JA groups (B) based on the COG database, and between JM and JA groups based on the pathways database (C).


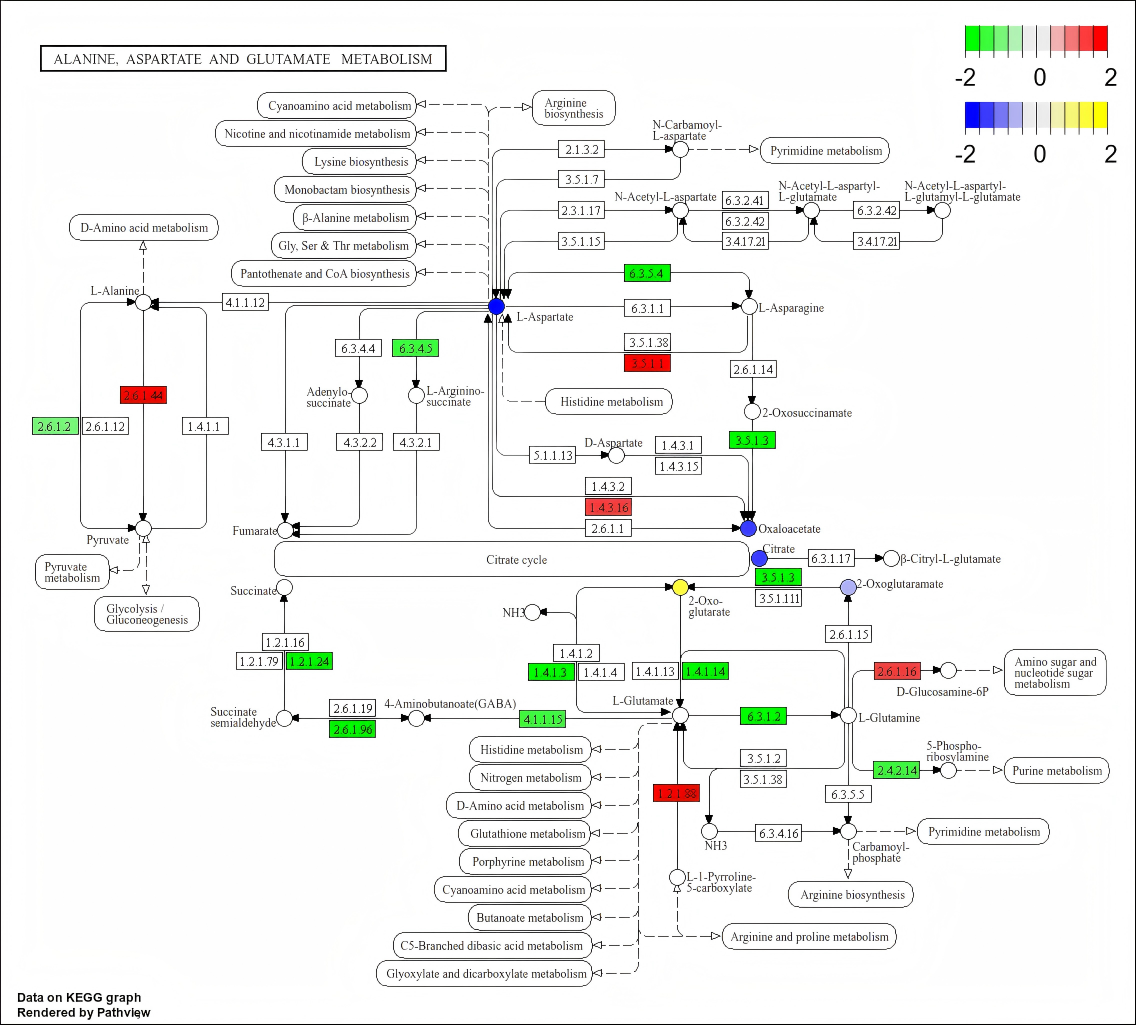


**Figure S6** Metabolic pathways of alanine, aspartate, and glutamate. Green boxes indicate significantly downregulated differentially expressed genes; red boxes indicate significantly upregulated differentially expressed genes. Blue circles indicate significantly downregulated differentially expressed metabolites; yellow circles indicate significantly upregulated differentially expressed metabolites.
